# Association of systemic inflammatory response index with ST segment elevation myocardial infarction and degree of coronary stenosis: a cross-sectional study

**DOI:** 10.1186/s12872-024-03751-z

**Published:** 2024-02-09

**Authors:** Jiongchao Guo, Yating Huang, Lamei Pang, Yuan Zhou, Jingjing Yuan, Bingfeng Zhou, Minmin Fu

**Affiliations:** 1 Department of Cardiology, The Third Affiliated Hospital of Anhui Medical University (The First People’s Hospital of Hefei), Hefei, 230000 Anhui China; 2 Department of Endocrinology, The Third Affiliated Hospital of Anhui Medical University (The First People’s Hospital of Hefei), Hefei, 230000 Anhui China; 3 Department of Endocrinology, Hefei BOE Hospital, Hefei, 230000 Anhui China; 4 Department of Cardiology, Hefei BOE Hospital, Hefei, 230000 Anhui China

**Keywords:** STEMI, Gensini score, Acute myocardial infarction, SIRI, Systemic inflammatory response index, NLR, Neutrophil to lymphocyte ratio

## Abstract

**Background:**

Systemic Inflammatory Response Index (SIRI), a composite inflammatory marker encompassing neutrophils, monocytes, and lymphocytes, has been recognized as a reliable marker of systemic inflammation. This article undertakes an analysis of clinical data from ST-segment Elevation Myocardial Infarction (STEMI) patients, aiming to comprehensively assess the relationship between SIRI, STEMI, and the degree of coronary stenosis.

**Methods:**

The study involved 1809 patients diagnosed with STEMI between the years 2020 and 2023. Univariate and multivariate logistic regression analyses were conducted to evaluate the risk factors for STEMI. Receiver operating characteristic (ROC) curves were generated to determine the predictive power of SIRI and neutrophil-to-lymphocyte ratio (NLR). Spearman correlation analysis was performed to assess the correlation between SIRI, NLR, and the Gensini score (GS).

**Results:**

Multivariate logistic regression analysis showed that the SIRI was the independent risk factor for STEMI (adjusted odds ratio (OR) in the highest quartile = 24.96, 95% confidence interval (CI) = 15.32–40.66, *P* < 0.001). In addition, there is a high correlation between SIRI and GS (β:28.54, 95% CI: 24.63–32.46, *P* < 0.001). The ROC curve analysis was performed to evaluate the predictive ability of SIRI and NLR for STEMI patients. The area under the curve (AUC) for SIRI was 0.789. The AUC for NLR was 0.754. Regarding the prediction of STEMI in different gender groups, the AUC for SIRI in the male group was 0.771. The AUC for SIRI in the female group was 0.807. Spearman correlation analysis showed that SIRI exhibited a stronger correlation with GS, while NLR was lower (SIRI: *r* = 0.350, *P* < 0.001) (NLR: *r* = 0.313, *P* < 0.001).

**Conclusion:**

The study reveals a strong correlation between the SIRI and STEMI as well as the degree of coronary artery stenosis. In comparison to NLR, SIRI shows potential in predicting acute myocardial infarction and the severity of coronary artery stenosis. Additionally, SIRI exhibits a stronger predictive capability for female STEMI patients compared to males.

## Introduction

Cardiovascular disease has emerged as a leading cause of global morbidity and mortality. Acute myocardial infarction (AMI) stands out as the predominant contributor to the morbidity and mortality associated with cardiovascular diseases (CVDs) worldwide. Based on NHANES data from 2017 to 2020, the overall prevalence of myocardial infarction (MI) among US adults aged 20 and above is 3.2%. Notably, in 2020, the United States witnessed approximately 109,199 fatalities attributed to myocardial infarction [1]. While chest pain represents a common symptom in most AMI cases, approximately 30% of patients do not manifest typical chest pain. Moreover, the detection of electrocardiograph (ECG) ST-segment elevation or depression only occurs during the onset, making it challenging to rely solely on coronary angiography (CAG), especially in regions with limited medical resources [2]. Consequently, the prevalence of misdiagnosis and failure to diagnose myocardial infarction remains substantial, while the prognosis of STEMI is closely intertwined with timely early diagnosis and intervention [3].

AMI predominantly stems from vascular atherosclerosis [4, 5]. The pathogenesis of vascular atherosclerosis is highly intricate, with inflammation playing a pivotal role in its onset and progression [5–10]. Previous studies have highlighted the association of composite inflammatory indicators, such as NLR, with the occurrence and development of STEMI [11–13]. SIRI, a composite inflammatory marker encompassing neutrophils, monocytes, and lymphocytes, has been recognized as a reliable surrogate marker of systemic inflammation, demonstrating its utility in predicting the long-term prognosis of acute coronary syndrome, contrast-induced nephropathy following Percutaneous Coronary Intervention (PCI), and new-onset atrial fibrillation [14–17]. Amidst inflammation, neutrophils and monocytes are recruited to inflamed tissues, participating in the response, and triggering the activation of lipid-laden macrophages, whereas lymphocytes play a protective role in atherosclerosis. Earlier research has established NLR as an independent risk factor for myocardial infarction, with predictive value for the prognosis of AMI patients. Currently, multiple scoring systems have emerged for predicting the prognosis of STEMI patients [18, 19]. However, existing studies on SIRI and STEMI predominantly focus on the correlation between SIRI and post-PCI complications, with limited exploration of the association between SIRI, STEMI, and coronary artery stenosis. Therefore, this study aimed to investigate the association of SIRI with STEMI and the degree of coronary stenosis and to compare SIRI with NLR to evaluate the difference between these two indicators on the correlation between STEMI and the degree of coronary stenosis. Consequently, the present study aims to delve into the association of SIRI with STEMI and the degree of coronary stenosis, and to draw comparisons between SIRI and NLR to discern disparities in their implications for the interplay between STEMI and the degree of coronary stenosis.

This article undertakes an analysis of clinical data from STEMI patients, aiming to comprehensively assess the relationship between SIRI, STEMI, and the degree of coronary stenosis, to provide clinical evidence for the prevention and treatment of STEMI.

## Methods

### Study participants

This study consecutively included 1809 hospitalized patients presenting to the Third Affiliated Hospital of Anhui Medical University with chest pain. Among them, 1258 patients with STEMI were selected as the experimental group, and 551 subjects without myocardial infarction and coronary artery stenosis of less than 30% by transcatheter CAG were selected as the control group. After the patients were enrolled, we conducted a power analysis, which revealed a power value greater than 0.8. This indicates that the sample included in this study is highly reliable. STEMI was diagnosed according to the European Society of Cardiology /ACC/AHA/World Federation of Heart Disease (EACS/AHA/WHF) “Global Definition of the Fourth Myocardial Infarction”. Exclusion criteria: (1) under 18 years old, unable to cooperate with patients. (2) patients who had recently taken lipid-lowering drugs. (3) patients with severe hepatic and renal insufficiency and hereditary hyperlipidemia. This study was carried out in accordance with the World Medical Association’s Code of Ethics (Helsinki Declaration) and approved by the Institutional Review Boards of the Third Affiliated Hospital of Anhui Medical University. All subjects provided written informed consent.

### Data collection

Baseline demographic and clinical data were meticulously procured for all patients, encompassing parameters such as age, gender, Body Mass Index (BMI), smoking history, hypertension history, alcohol consumption history, diabetes history, Neutrophil count, monocyte count, lymphocyte count, Red Blood Cell count (RBC), Total Cholesterol (TC), Triglycerides (TG), Low-Density Lipoprotein Cholesterol (LDL-C), and High-Density Lipoprotein Cholesterol (HDL-C), among others.

### Definitions

SIRI was defined as neutrophils × monocytes / lymphocytes;

NLR was defined as neutrophils / lymphocytes.

### Statistical analysis

IBM SPSS Statistics 26.0 (IBM, New York, NY, USA) and R version 4.2.2 (R Foundation for Statistical Computing, University of Science and Technology of China) were utilized for the statistical analyses in this study. Continuous variables were presented as mean ± standard deviation or as median (interquartile range). A comparison of data between the two groups was conducted using the T-test or the Mann-Whitney U test, depending on the normality of the data. Categorical variables were presented as counts and percentages, and statistical analysis was performed using the Chi-square test or Fisher’s exact test. Univariate and multivariate logistic regression analyses were conducted to assess the association of SIRI with STEMI and the Gensini Score. ROC curves were constructed to ascertain the cut-off values and predictive efficacy of SIRI and NLR. Spearman correlation analysis was executed to evaluate the relationship between SIRI, NLR, and the Gensini Score. All statistical tests were two-tailed, and a significance threshold of *P* < 0.05 was deemed statistically significant.

## Results

### Baseline characteristics

A comprehensive comparative analysis of the general clinical data between the two cohorts revealed the following key observations: Notably, no significant discrepancies were discerned in terms of lymphocyte count, BMI, and TG levels between the STEMI group and the control group (*P* > 0.05) (Table 1). Conversely, the prevalence of male individuals, smokers, hypertensive individuals, and diabetic individuals within the STEMI group exhibited a notably heightened prevalence compared to the control group (*P* < 0.05) (Table 1). Furthermore, the STEMI group exhibited significantly elevated values in terms of age, neutrophil count, lymphocyte count, monocyte count, TC, LDL-C, SIRI, and NLR in comparison to the control group. Conversely, the HDL-C levels in the control group were significantly higher than those in the STEMI group (*P* < 0.05) (Table 1).

**Table 1:** Baseline demographic and clinical characteristics of the study population according to STEMI

	Non-STEMI group (*N* = 551)	STEMI group (*N* = 1258)	*P*
Age (years)	59.16 ± 11.32	62.22 ± 13.52	< 0.001*
Sex (male)	340 (61.71%)	968 (76.95%)	< 0.001*
BMI (Kg/m^2^)	23.90 ± 2.85	23.96 ± 2.85	0.673
Hypertension	226 (41.02%)	715 (56.84%)	< 0.001*
Diabetes	98 (17.79%)	444 (35.29%)	< 0.001*
Smoking	132 (23.96%)	681 (54.13%)	< 0.001*
Neutrophil (×10^9^)	4.09 ± 1.68	6.83 ± 3.10	< 0.001*
Lymphocyte (×10^9^)	1.84 ± 0.69	1.78 ± 0.69	0.073
Monocyte (×10^9^)	0.44 ± 0.17	0.61 ± 0.29	< 0.001*
Red blood cell (×10^12^)	4.34 ± 0.57	4.39 ± 0.66	0.153
TC(mmol/L)	4.12 ± 1.07	4.44 ± 0.95	< 0.001*
TG (mmol/L)	1.63 ± 1.33	1.67 ± 1.12	0.556
HDL-C (mmol/L)	1.20 ± 0.40	0.97 ± 0.24	< 0.001*
LDL-C (mmol/L)	2.27 ± 0.89	2.70 ± 0.83	< 0.001*
SIRI	0.86 (0.55,1.49)	2.03 (1.26,3.38)	< 0.001*
NLR	2.10 (1.54,2.97)	3.51 (2.51,5.56)	< 0.001*

Abbreviations: HDL-C, High-density lipoprotein cholesterol; LDL-C, Low-density lipoprotein cholesterol; TC, triglyceride; TG, triglyceride; SIRI, Systemic Inflammatory Response Index; NLR, Neutrophil-to-Lymphocyte Ratio

### Univariate and multivariate logistic regression analysis

The results of the univariate regression analysis suggested a potential correlation between the SIRI and the NLR as risk factors for STEMI (Table 2). To demonstrate the association of SIRI with STEMI, all patients were divided into four groups according to the quartile values of SIRI (Q1: SIRI ≤ 0.87, Q2: SIRI 0.87–1.60, Q3: SIRI 1.60–2.86, Q4: SIRI > 2.86). The OR value of STEMI in the highest quartile compared with the lowest quartile was 22.74 (95% CI: 15.00-34.47, *P* < 0.001) (Table 3). After adjusting for gender, age, BMI, hypertension, diabetes, smoking, TC, TG, HDL-C and LDL-C, the risk of STEMI remained significantly higher in the highest quartile than in the lowest quartile (OR: 24.96, 95% CI: 15.32–40.66, *P* < 0.001) (Table 3). Finally, after further adjusting for the above confounding factors, the results of a trend analysis suggest that the trend of an increasing probability of STEMI occurrence with rising SIRI holds statistical significance (OR: 2.70, 95% CI: 2.36–3.09, *P* < 0.001) (Table 3).

**Table 2:** Univariate logistic regression analysis of selected variables on STEMI

	OR (95%CI)	*P*
Age	1.02 (1.01, 1.03)	< 0.001
Sex	2.07 (1.67, 2.57)	< 0.001
BMI	0.95 (0.92, 0.98)	0.002
Hypertension	1.89 (1.55, 2.32)	< 0.001
Diabetes	2.52 (1.97, 3.23)	< 0.001
Smoking	3.75 (2.99, 4.69)	< 0.001
Neutrophil	2.00 (1.85, 2.17)	< 0.001
Monocyte	34.92(19.91,61.24)	< 0.001
TC	1.42 (1.27, 1.58)	< 0.001
HDL-C	0.07 (0.05, 0.11)	< 0.001
LDL-C	1.92 (1.67, 2.19)	< 0.001
SIRI	2.53 (2.22, 2.89)	< 0.001
NLR	1.59 (1.48, 1.72)	< 0.001

Abbreviations: TC, triglyceride; HDL-C, High-density lipoprotein cholesterol; LDL-C, Low-density lipoprotein cholesterol; SIRI, Systemic Inflammatory Response Index; NLR, Neutrophil-to-Lymphocyte Ratio

**Table 3:** Multivariate logistic analysis to determine associations between SIRI subgroup levels and STEMI

	Model 1		Model 2		Model 3	
	OR (95%CI)	*P*	OR (95%CI)	*P*	OR (95%CI)	*P*
SIRI	2.53 (2.22, 2.89)	< 0.001	2.42 (2.13, 2.76)	< 0.001	2.36 (2.05, 2.72)	< 0.001
SIRI quartile						
SIRI ≤ 0.87	1.0		1.0		1.0	
0.87 ≤ SIRI ≤ 1.60	3.03 (2.31, 3.97)	< 0.001	2.77 (2.10, 3.64)	< 0.001	2.43 (1.76, 3.37)	< 0.001
1.60 ≤ SIRI ≤ 2.86	6.96 (5.14, 9.42)	< 0.001	6.29 (4.63, 8.55)	< 0.001	5.60 (3.91, 8.02)	< 0.001
2.86 ≤ SIRI	22.74 (15.00, 34.47)	< 0.001	20.53 (13.50, 31.25)	< 0.001	24.96 (15.32, 40.66)	< 0.001
SIRI for trend	2.74 (2.44, 3.08)	< 0.001	2.64 (2.35, 2.96)	< 0.001	2.70 (2.36, 3.09)	< 0.001

Model 1 Non-adjusted

Model 2 adjust for: sex, age

Model 3 adjust for: sex, age, hypertension, diabetes, smoking, TC, TG, HDL-C, LDL-C, BMI

Abbreviations: TC, triglyceride; TG, total cholesterol; HDL-C, High-density lipoprotein cholesterol; LDL-C, Low-density lipoprotein cholesterol; SIRI, Systemic Inflammatory Response Index; BMI, Body Mass Index

To demonstrate the association of SIRI with Gensini Score, all patients were divided into four groups according to the quartile values of NHR (Q1: SIRI ≤ 0.87, Q2: SIRI 0.87–1.60, Q3: SIRI 1.60–2.86, Q4: SIRI > 2.86). The β value of GS in the highest quartile compared with the lowest quartile was 37.97 (95% CI: 33.76–42.18, *P* < 0.001) (Table 4). After adjusting for gender, age, smoking, diabetes, hypertension, TC, TG, HDL-C, LDL-C, the risk of high GS remained significantly higher in the highest quartile than in the lowest quartile (β:28.54, 95% CI: 24.63–32.46, *P* < 0.001) (Table 4). After further adjusting for the above confounding factors, the results of a trend analysis suggest that the trend of an increasing GS with rising SIRI holds statistical significance (β: 9.13, 95% CI: 7.90–10.35, *P* < 0.001) (Table 4).

**Table 4:** Multivariate linear regression analysis to determine associations between SIRI subgroup levels and Gensini score

	Model 1		Model 2		Model 3	
	**β** (95%CI)	*P*	**β**(95%CI)	*P*	**β**(95%CI)	*P*
SIRI	2.65 (2.24, 3.07)	< 0.001	2.65 (2.23, 3.06)	< 0.001	2.26 (1.91, 2.62)	< 0.001
SIRI quartile						
SIRI ≤ 0.87	0		0		0	
0.87 ≤ SIRI ≤ 1.60	18.29 (13.85, 22.73)	< 0.001	18.20 (13.75, 22.65)	< 0.001	13.78 (9.83, 17.72)	< 0.001
1.60 ≤ SIRI ≤ 2.86	29.58 (25.28, 33.87)	< 0.001	29.53 (25.21, 33.86)	< 0.001	22.13 (18.24, 26.01)	< 0.001
2.86 ≤ SIRI	37.97 (33.76, 42.18)	< 0.001	37.96 (33.71, 42.20)	< 0.001	28.54 (24.63, 32.46)	< 0.001
SIRI for trend	12.15 (10.84, 13.46)	< 0.001	12.14 (10.82, 13.46)	< 0.001	9.13 (7.90, 10.35)	< 0.001

Model 1 Non-adjusted

Model 2 adjust for: sex, age

Model 3 adjust for: sex, age, hypertension, diabetes, smoking, TC, TG, HDL-C, LDL-C, BMI

Abbreviations: TC, triglyceride; TG, total cholesterol; HDL-C, High-density lipoprotein cholesterol; LDL-C, Low-density lipoprotein cholesterol; SIRI, Systemic Inflammatory Response Index; BMI, Body Mass Index

### Subgroup analyses

**Fig. 1:**
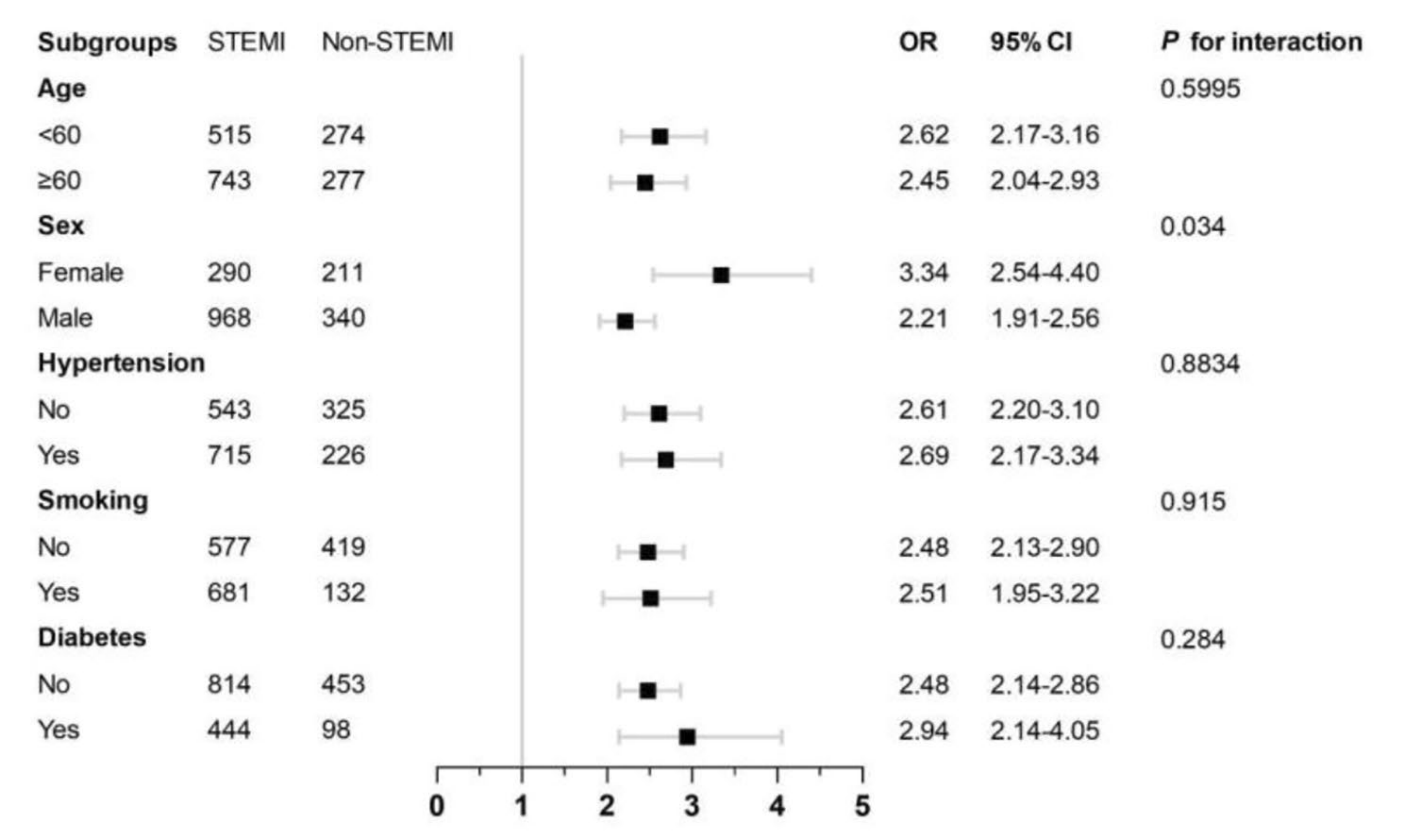
Stratified logistic regression analysis model to explore variables affecting the correlation between SIRI and STEMI. Above model adjusted for age (≤ 60 vs. > 60), sex (female vs. male), smoking, diabetes, hypertension. Abbreviations: SIRI, Systemic Inflammatory Response Index

Subgroup analyses unveiled that age (< 60 vs. ≥60), sex (female vs. male), smoking, and diabetes, as well as hypertension, did not significantly influence the relationship between SIRI and STEMI. The tests evaluating the interaction of age (< 60 vs. ≥60), smoking, diabetes, and hypertension with respect to the effects of SIRI on STEMI did not yield statistically significant results (P for interaction > 0.05) (Fig. 1). Notably, sex (female vs. male) demonstrated a significant influence on the interaction between SIRI and STEMI, suggesting potential gender-based disparities in the impact of SIRI on STEMI occurrence (Fig. 1).

### ROC curve analysis

**Fig. 2:**
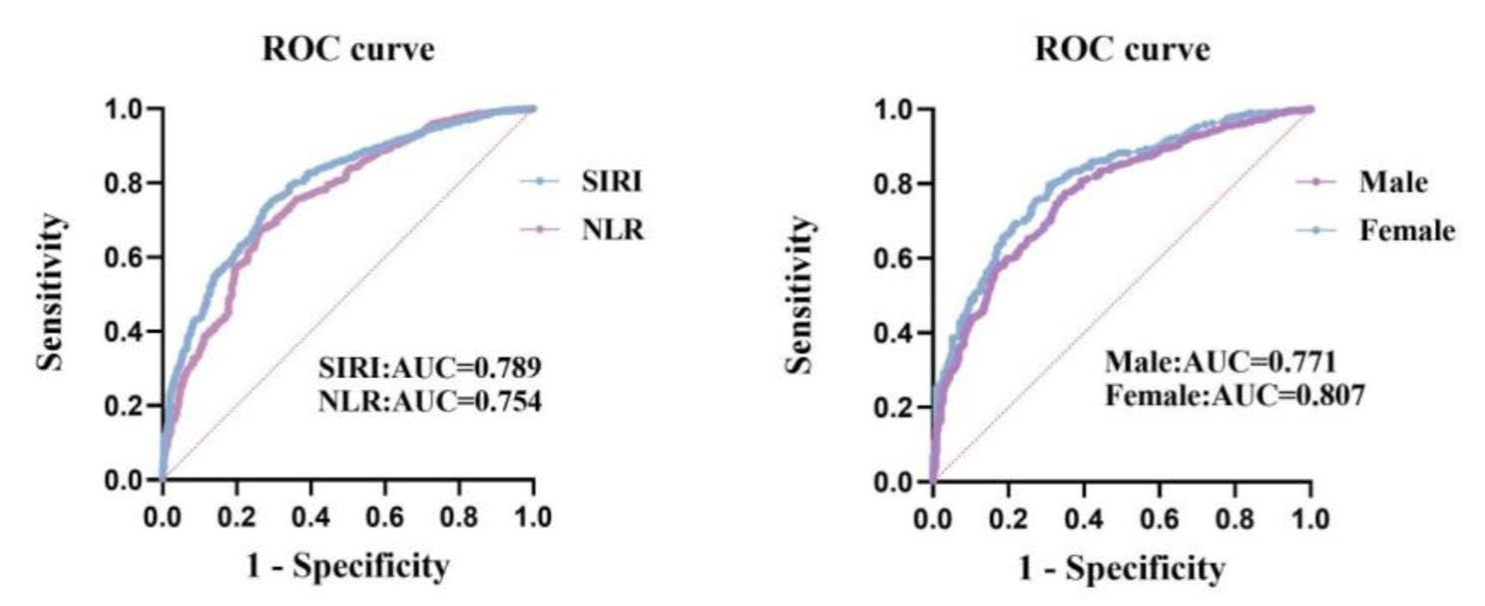
(**A**) ROC curve of SIRI and NLR predicting the risk of STEMI. (**B**) ROC curve of male and female predicting the risk of STEMI. Abbreviations: ROC curve, receiver operator characteristic curve; AUC, area under the curve; SIRI, Systemic Inflammatory Response Index; NLR, Neutrophil-to-Lymphocyte Ratio; STEMI, St-segment Elevation Myocardial Infarction

The ROC curve analysis was performed to evaluate the predictive ability of SIRI and NLR for STEMI. The results are as follows: SIRI: The AUC for SIRI was 0.789 with a 95% CI of (0.767, 0.811) (the specificity was 70.6%, the sensitivity was 75.1%, and the cut-off value was 1.257) (Table 5) (Fig. 2). NLR: The AUC for NLR was 0.754 with a 95% CI of (0.730, 0.778) (the specificity was 73.3%, the sensitivity was 67.5%, and the cut-off value was 2.853) (Table 5) (Fig. 2).

**Table 5:** ROC curve of SIRI and NLR predicting for STEMI.

	AUC	95%CI	Youden index	Specificity	Sensitivity	PPV	NPV	Positive likelihood ratio	Negative likelihood ratio	Diagnostic odds
SIRI	0.789	0.767–0.811	1.257	0.706	0.751	0.854	0.554	2.554	0.353	7.235
NLR	0.754	0.730–0.778	2.853	0.733	0.675	0.852	0.497	2.528	0.443	5.707

Abbreviations: ROC curve, receiver operator characteristic curve; AUC, area under the curve; SIRI, Systemic Inflammatory Response Index; NLR, Neutrophil-to-Lymphocyte Ratio; PPV, positive predictive value; NPV, negative predictive value

The ROC curves of SIRI for STEMI were plotted separately for men and women. The results are as follows: Female: The AUC for SIRI was 0.807 with a 95% CI of (0.769, 0.845) (the specificity was 79.7%, the sensitivity was 69.2%, and the cut-off value was 1.026) (Table 6) (Fig. 2). Male: The AUC for SIRI was 0.771 with a 95% CI of (0.743, 0.799) (the specificity was 65.0%, the sensitivity was 77.5%, and the cut-off value was 1.258) (Table 6) (Fig. 2).

**Table 6:** ROC Curve of SIRI divided into female and male predicting for STEMI.

SIRI	AUC	95%CI	Youden index	Specificity	Sensitivity	PPV	NPV	Positive likelihood ratio	Negative likelihood ratio	Diagnostic odds
Female	0.807	0.769–0.845	1.026	0.692	0.797	0.780	0.712	2.588	0.293	8.833
Male	0.771	0.743–0.799	1.258	0.650	0.775	0.863	0.503	2.214	0.346	6.399

Abbreviations: ROC curve, receiver operator characteristic curve; AUC, area under the curve; SIRI, Systemic Inflammatory Response Index; NLR, Neutrophil-to-Lymphocyte Ratio; PPV, positive predictive value; NPV, negative predictive value

These findings indicate that in comparison to the NLR, SIRI possesses a higher diagnostic predictive value. Moreover, SIRI might demonstrate a relatively superior predictive capacity for women compared to men.

### Correlation of SIRI, NLR and GS

**Fig. 3:**
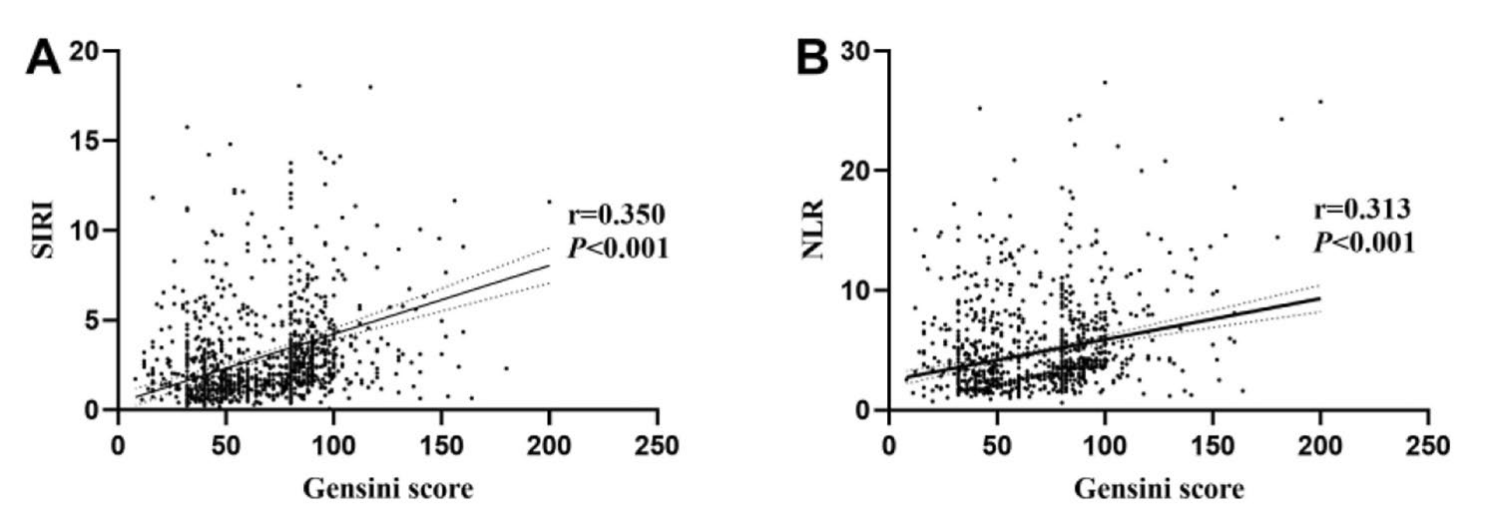
(**A**) Correlation of SIRI with Gensini score (Spearman correlation analysis). (**B**) Correlation of NLR with Gensini score (Spearman correlation analysis). Abbreviations: SIRI, Systemic Inflammatory Response Index; NLR, Neutrophil-to-Lymphocyte Ratio

Spearman correlation analysis was executed to assess the relationship between the SIRI, the NLR, and the GS. The findings are as follows: SIRI demonstrated a substantial correlation with GS (*r* = 0.350, *P* < 0.05) (Table 7) (Fig. 3), denoting a moderate positive correlation between SIRI and the severity of coronary artery lesions. NLR exhibited a relatively lower correlation with GS compared to SIRI (*r* = 0.315, *P* < 0.05) (Table 7) (Fig. 3).

**Table 7:** Correlation of SIRI and NLR with Gensini score

	r	*P*
SIRI	0.350	< 0.001
NLR	0.313	< 0.001

Abbreviations: SIRI, Systemic Inflammatory Response Index; NLR, Neutrophil-to-Lymphocyte Ratio

## Discussion

This study has discovered a strong correlation between SIRI and STEMI, as well as the degree of coronary artery stenosis. In comparison to the NLR, SIRI demonstrates promising potential in predicting acute myocardial infarction and the severity of coronary artery stenosis. Furthermore, it exhibits a higher predictive capability for female STEMI patients than for males. STEMI primarily stems from thrombosis precipitated by the rupture of coronary atherosclerotic plaques, which are incited by processes of inflammation and aberrant lipid metabolism [5, 20–23]. Various inflammatory markers play crucial roles in the onset and progression of atherosclerosis. Previous studies have emphasized the association between elevated white blood cell count and the occurrence and unfavorable prognosis of coronary heart disease [24, 25]. For example, neutrophil count and monocyte count are related to the risk of coronary heart disease [26, 27]. However, a single inflammatory marker can only provide insights into one aspect of the inflammatory process and may not accurately predict the extent of inflammation. SIRI combines three inflammatory biomarkers: neutrophils, monocytes, and lymphocytes. It offers the advantages of comprehensiveness, ease of acquisition, and cost-effectiveness. Previous research has demonstrated its correlation with the prognosis of cardiovascular disease to some degree, particularly in terms of its potential unique value for predicting the prognosis of AMI patients [28]. Yan Chen et al. developed a simple predictive model for the prognosis of elderly hospitalized patients based on SIRI [19]. In addition, another study has indicated that AMI patients with high SIRI may have a higher incidence of MACE [29]. Nevertheless, the correlation between SIRI and STEMI diagnosis, as well as the severity of coronary artery stenosis, has not been evaluated in previous research. Further investigation is needed to explore this association, and this article aims to analyze the relationship between SIRI and STEMI, as well as the degree of coronary stenosis. This article analyzes the relationship between SIRI and STEMI, as well as the degree of coronary stenosis. The results indicate that both SIRI and NLR are independent risk factors for STEMI, with SIRI exhibiting a higher predictive value for STEMI than NLR. Additionally, SIRI shows a significant correlation with the Gensini score, suggesting its potential utility in predicting the severity of coronary artery stenosis.

Atherosclerosis represents a multifaceted process, entailing intricate interplay between lipids and immune-inflammatory cells. Research has elucidated that substances derived from inflammatory cells possess the intrinsic ability to incite activation of the coagulation system, even in the absence of conventional risk factors. Concurrently, inflammatory cell infiltration within atherosclerotic lesions instigates cytokine cascades [30]. Simultaneously, inflammation can foster plaque destabilization and stimulate plaque rupture, culminating in the onset of acute coronary syndrome [31]. This underscores the pivotal role of dysregulated inflammation in the mechanism of thrombosis [32].

The foam cells, which constitute the atherosclerotic plaque, primarily originate from monocytes that migrate to the arterial wall and differentiate into macrophages, engulfing oxidized low-density lipoprotein cholesterol (OX-LDL-C) [7]. Monocytes have the ability to migrate into the vascular endothelium, where they transform into macrophages and express scavenger receptors crucial for the uptake of OX-LDL-C. Upon the engulfment of OX-LDL-C and other lipids, monocytes develop into foam cells, depositing within the intima of blood vessels to form atherosclerotic plaques, a significant contributor to the development of coronary atherosclerosis. Moreover, monocytes promote the destabilization of the fibrous cap and participate in plaque rupture through the secretion of enzymes [33]. Additionally, monocytes are involved in thrombus formation, leading to a cascade of coagulation reactions [34]. Previous research has highlighted that levels of circulating pro-inflammatory monocytes and monocyte-platelet complexes are inversely correlated with myocardial viability and left ventricular functional recovery assessed by magnetic resonance imaging post-AMI [35, 36]. Furthermore, inhibiting monocyte infiltration and differentiation can attenuate early-stage atherosclerosis [37, 38]. Numerous therapeutic interventions have demonstrated the protection of the post-AMI myocardium by facilitating the transition of monocytes/macrophages from a pro-inflammatory phenotype to an anti-inflammatory phenotype [39–41].

Although previously underappreciated in cardiovascular research, the past decade has illuminated the pivotal regulatory role of neutrophils in cardiovascular inflammation. Neutrophils, active participants in all phases of atherosclerosis, have emerged as a crucial target for the development of therapeutics addressing cardiovascular diseases [42]. Neutrophils play a critical role in the inflammatory response of atherosclerosis, potentially releasing numerous inflammatory mediators, chemokines, and reactive oxygen species, inducing endothelial cell injury and subsequent myocardial tissue ischemia [43, 44]. Studies have demonstrated a positive correlation between the extent of hypercholesterolemia-induced neutrophilia and the formation of early atherosclerotic lesions, which can influence atherosclerotic plaques and provoke thrombotic complications associated with atherosclerosis [45, 46]. Neutrophils release decondensed nucleosomes known as neutrophil extracellular traps (NETs), which induce platelet activation and initiate coagulation. Furthermore, the secretion of various pro-coagulant granzymes by neutrophils collectively contributes to thrombosis formation and subsequent AMI [47].

Research has indicated that elevated neutrophil counts represent an independent predictive factor for adverse cardiovascular events [48]. In the context of AMI, neutrophils are recruited to the infarcted myocardium within the initial hours to initiate localized inflammation and tissue damage. Unfortunately, neutrophils also exacerbate myocardial tissue destruction by releasing myeloperoxidase (MPO) and reactive oxygen species (ROS), both of which are associated with poor prognosis in AMI patients [49]. In addition, neutrophil-derived mediators regulate the functions of monocytes, macrophages, and dendritic cells, including recruitment, phagocytic capacity, and cytokine release [50, 51]. Previous investigations have demonstrated a high abundance of neutrophils in ruptured carotid plaques, this suggests that neutrophils are closely related to the formation of unstable plaque rupture and that it may play a role in plaque destabilization [52, 53].

Distinct from the roles of neutrophils and monocytes, lymphocytes are known to play a regulatory function in atherogenic inflammation, potentially exerting an inhibitory effect on atherosclerosis [54]. A lower lymphocyte count has been associated with an increased risk of mortality due to heart failure and coronary artery-related disease [55]. B lymphocytes are capable of modulating the release of pro-inflammatory cytokines such as IL-1β, TNF, and IL-18 through the release of CD20 antibody or B cell activating factor (BAFF), subsequently reducing the systemic inflammatory response, diminishing myocardial infarction size, and enhancing cardiac function post-myocardial infarction [56]. Additionally, the infiltration of regulatory T lymphocytes (Tregs) in the myocardial infarction area has demonstrated some favorable effects in reducing infarction size and preventing adverse left ventricular remodeling [57]. However, during the occurrence of STEMI, the population of Tregs is significantly diminished. This reduction in Tregs can be attributed to the accumulation of Tregs in the area of myocardial infarction, a decreased capacity of the thymus to produce Tregs, and an increased rate of Treg apoptosis following AMI. Tregs can inhibit the recruitment of inflammatory cells, such as neutrophils, monocytes, and CD4 + T lymphocytes, and suppress the local expression of pro-inflammatory cytokines like TNF-α and IL-1β [57, 58]. What’s more, it also can promote the polarization of macrophages to an anti-inflammatory M2 phenotype, and inhibit polarization to a pro-inflammatory M1 phenotype [59]. These actions collectively inhibit the development of atherosclerosis and myocardial remodeling following myocardial infarction.

In the context of this study, regression analysis and subgroup analysis, following the adjustment of risk factors, revealed a strong correlation between the SIRI and STEMI, as well as the extent of coronary artery stenosis. This association suggests the potential of SIRI in predicting acute myocardial infarction and the degree of coronary artery stenosis, thus facilitating the early diagnosis of STEMI and enabling timely interventions, such as thrombolysis or Percutaneous Transluminal Coronary Intervention (PCI).

Primarily, in evaluating the diagnostic efficacy of SIRI for STEMI, it was observed that the diagnostic efficacy of this marker for STEMI may vary based on gender, with higher diagnostic efficacy observed in women. Considering the comparatively low incidence of STEMI in women, especially premenopausal women, owing to the protective effects of estrogen, the diagnostic capacity of SIRI for STEMI may offer critical insights. Clinicians may tend to overlook STEMI occurrences in this demographic, and the application of SIRI can aid in improved diagnosis among this population. Moreover, given the limitations associated with the specificity of ECG, the requirement for specialized equipment for troponin acquisition, the time delay of 3–6 h for troponin elevation, and its significantly higher cost compared to routine blood tests, the economic feasibility and ease of acquisition of SIRI from routine blood tests make it readily deployable in clinical settings. This underscores its potential to enable early detection of MI. This enables healthcare professionals to promptly establish a definitive diagnosis of STEMI and proceed with thrombolysis or interventional treatment for the patients [60, 61].

Furthermore, this study demonstrated that compared to the NLR, SIRI exhibited a stronger correlation with the degree of coronary stenosis. This finding can aid in the early stratification of risk factors in STEMI patients. Particularly in resource-constrained medical settings where prompt access to CAG may not be feasible and where many patients refuse invasive procedures, the use of SIRI, an economical and straightforward index, to assess the extent of coronary stenosis could enable timely evaluation of patients’ coronary artery conditions and risk stratification. This has the potential to yield significant benefits for clinical practice.

In short, in clinical practice, early diagnosis of STEMI mainly relies on cardiac enzymes, troponin, ECG, and CAG. In the early stages of the disease, cardiac enzymes and troponin may not be elevated, and the specificity of ECG is relatively low. Additionally, many remote areas may not have timely access to these laboratory tests and CAG, which can lead to delays in diagnosing STEMI and subsequently formulating appropriate treatment plans, adversely affecting patient outcomes. Therefore, SIRI, as an easily accessible indicator, may serve as an adjunct diagnostic tool to enhance the diagnostic process of STEMI. This can facilitate timely diagnosis of STEMI, allowing prompt thrombolysis or coronary intervention, thereby enabling early intervention to improve patient prognosis. On the other hand, since the gold standard for assessing the degree of coronary artery stenosis relies on CAG, which is an invasive procedure, many patients are reluctant to undergo it. SIRI, as an easily obtainable composite indicator, may be correlated with the severity of coronary artery stenosis. Clinicians can use this indicator to roughly assess the extent of coronary artery stenosis, thereby better predicting cardiovascular risk and making timely adjustments to subsequent treatment plans. This has the potential to improve patient prognosis to a certain extent.

## Limitation

Unfortunately, the present study has some limitations that require attention and rectification. Firstly, it was a cross-sectional investigation with a restricted sample size and a limited range of included variables, potentially leaving it vulnerable to confounding biases and limiting the strength of the evidence provided. The distribution of confounders demonstrated homogeneity among the groups, with no significant disparities observed. Secondly, the selection of recruitment sites exclusively from one hospital may introduce selection bias, resulting in an underrepresented sample. Furthermore, the accuracy and generalizability of the SIRI should be evaluated through research involving larger samples. Consequently, future endeavors should involve a multi-center prospective cohort study with a more substantial sample size. Thirdly, our study was focused on the diagnosis of STEMI, emphasizing the need for prospective research to explore the prognosis of patients. Lastly, it’s worth noting that the laboratory data used to calculate SIRI in this study was obtained from a single measurement, which does not rule out the possibility of temporal variations and experimental errors. Therefore, future investigations may require repeated measurements at multiple time points to establish the reliability of this indicator.

## Future directions

Currently, research on SIRI is predominantly limited to single-center retrospective studies, which provide relatively weaker evidence. Prior to the clinical application of this indicator, it is crucial to conduct larger-scale multi-center prospective studies. These studies will enable the acquisition of high-quality evidence and establish the potential correlation between SIRI and STEMI or other cardiovascular diseases.

## Conclusion

The study reveals a strong correlation between the SIRI and STEMI as well as the degree of coronary artery stenosis. In comparison to NLR, SIRI shows potential in predicting acute myocardial infarction and the severity of coronary artery stenosis. Additionally, SIRI exhibits a stronger predictive capability for female STEMI patients compared to males.

## Data Availability

The datasets used and/or analysed during the current study are available from the corresponding author, Bingfeng Zhou, upon a reasonable request. The data are not publicly available due to their containing information that could compromise the privacy of patients.
